# Prognostic Variables and Surgical Management of Foot Melanoma: Review of a 25-Year Institutional Experience

**DOI:** 10.5402/2011/384729

**Published:** 2011-07-26

**Authors:** Omar M. Rashid, Julia C. Schaum, Luke G. Wolfe, Nooshin K. Brinster, James P. Neifeld

**Affiliations:** ^1^Department of Surgery, Virginia Commonwealth University and the Massey Cancer Center, Richmond, VA 23298-0645, USA; ^2^Department of Pathology, Virginia Commonwealth University and the Massey Cancer Center, Richmond, VA 23298-0662, USA

## Abstract

*Introduction*. Cutaneous foot melanoma is rare, challenging to manage, and not adequately examined in the literature. This study evaluated the prognostic variables and surgical management of foot melanoma. *Materials and Methods*. Foot melanoma cases managed at an academic center from 1985 to 2010 were retrospectively reviewed. *Results*. 46 patients were identified with a broad range of demographic characteristics. Overall recurrence was 32.6%: 19% acral lentiginous, 57% nodular, 66% superficial spreading, 30% melanoma unspecified, 50% severely atypical; 53% ulcerated, 23% nonulcerated; 29% on the dorsum of the foot, 17% heel, 60% ankle, 22% toe, 50% plantar; 0% <1 mm thick, 47% 1–4 mm, 33% >4 mm. 13 had positive nodes, 4 (31%) of whom recurred. Prognostic factors and recurrence did not correlate, and survival was 96% with a median followup of 91 months. *Conclusions*. Aggressive management of foot melanoma may result in excellent long-term survival even following disease recurrence.

## 1. Introduction

Malignant melanoma represents a major public health concern worldwide and the annual incidence has been steadily rising since 1935 [1]. Approximately 3–5% of all cutaneous melanomas arise in the foot, and foot melanoma poses a challenge to clinicians who must balance adequate oncologic resection with preservation of limb function [2]. When margins smaller than recommended have been performed to avoid amputation or skin grafting, the reported recurrence rates have also been many times higher than at other sites [3]. The literature has not adequately evaluated the prognostic variables that guide the surgical management of foot melanoma, as recommendations are based on studies on melanoma arising on the trunk and elsewhere on the extremities [4, 5]. 

Historically, clinicians have focused on clinical factors, histology, anatomic location, and the presence or absence of metastatic disease to determine the prognosis of patients with melanoma [3, 6]. Pathologic variables include Breslow thickness, Clark's level of invasion, mitotic index, presence of tumor infiltrating lymphocytes, ulceration, bleeding, and lymph node status [4, 5]. Because of the more vertical growth pattern of acral lentiginous and nodular melanomas compared to the more radial growth pattern of superficial spreading melanoma on pathology, it has been thought that superficial spreading melanoma confers a better prognosis [3]. Historically, in response to Paget's soil versus seed theory, Ewing argued that the anatomic location of a neoplasm determines prognosis because of the variable density of soft tissues, blood, and lymphatic vessels [3]. In the foot, this argument was even further applied to distinguish between the prognosis of melanoma arising on the dorsum of the foot versus the plantar aspect of the foot based on the variable thickness of the skin and the variable density of vascular and lymphatic networks between these sites [3]; however, there is no consensus in the literature regarding the variables that predict prognosis and, therefore, which should guide the management of foot melanoma. 

The literature does not adequately analyze the prognostic variables, management, and outcomes of foot melanoma. Because foot melanoma is so rare, many publications combine melanoma of the foot with the hand, leg, or thigh for statistical purposes. Even papers that focus solely on the foot are often confounding in their conclusions because they combine cutaneous melanoma with subungual melanoma. In addition, there has been great variability in the factors that the literature has analyzed, even in regard to such clinically significant considerations as pathology, thickness, lymph node status, metastasis, recurrence, and survival. This retrospective study was performed to review the prognostic variables, management, and outcomes of foot melanoma to try to arrive at surgical guidelines.

## 2. Materials and Methods

Patients treated for cutaneous melanoma of the foot from 1985 to 2010 at the Virginia Commonwealth University Health System were retrospectively reviewed for demographic data, histology, anatomic location, Breslow thickness, mitotic index, ulceration, operation, surgical margin, lymph node status, location of recurrence, disease-free survival, and overall survival. The Virginia Commonwealth University Institutional Review Board granted approval for this study, and this study complied with all guidelines for ethical research. Recurrence analysis was performed by calculating and plotting the median cumulative function using JMP8 software. Statistical analysis utilized logistic regression to evaluate for significance.

## 3. Results

46 patients were treated for foot melanoma from 1985 to 2010. Patient followup was a median of 91 months, a mean of 101 months, and ranged from 4 to 300 months. Patients were followed for 3 months for the first 2 years, then for 6 months until the 5th year, followed by yearly thereafter. No patients were lost to follow up. Patients were treated with wide local excision with primary closure or skin grafting; amputation was performed when a lesser procedure was unable to obtain an adequate margin and leave a functioning foot. Lymphadenectomy was performed when lymph node metastasis was detected clinically or by sentinel node biopsy. Palliative debulking of wound, regional, and distant recurrences was performed when appropriate. No patients received adjuvant therapy. 

**Figure 1:**
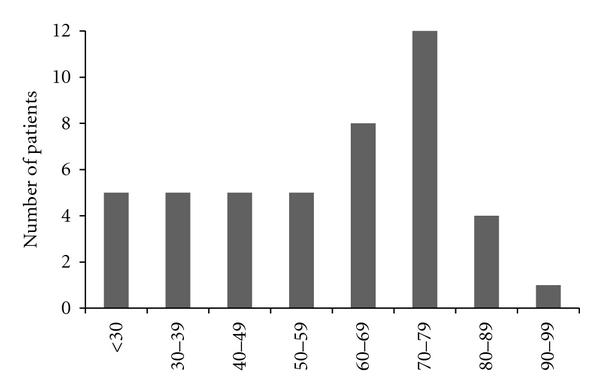
The demographics of the patients by age distribution.

 The patient demographics, pathology, site of origin, and surgical treatment were examined. Of the 46 patients, 15 were men and 31 were women. Age at diagnosis ranged from 16 to 99 years of age, with a median of 62 years (mean 59) (Figure 1). Ethnic distribution included 31 Caucasians, 12 African Americans, 1 Hispanic, and 2 others. The pathologic demographics included 16 acral lentiginous, 10 unspecified, 9 superficial spreading, 7 nodular, 2 severely atypical (melanoma which the pathologists could not further classify), and 2 desmoplastic; 16 were ulcerated with a median thickness of 2.38 mm (mean 4.9 mm) and a median mitotic index of 2 per ten high power fields (mean 4). Clark's level of invasion was not uniformly reported in these patients. The anatomic distribution included 17 on the dorsum of the foot, 6 heel, 5 ankle, 9 toe, 8 plantar, and 1 webspace. 39 patients underwent wide local excision, 6 toe amputation, 1 below knee amputation, and 20 underwent sentinel lymph node biopsy or lymph node dissection. The patients represented a broad distribution of demographic characteristics, pathologic subtypes, and sites of origin.

**Figure 2:**
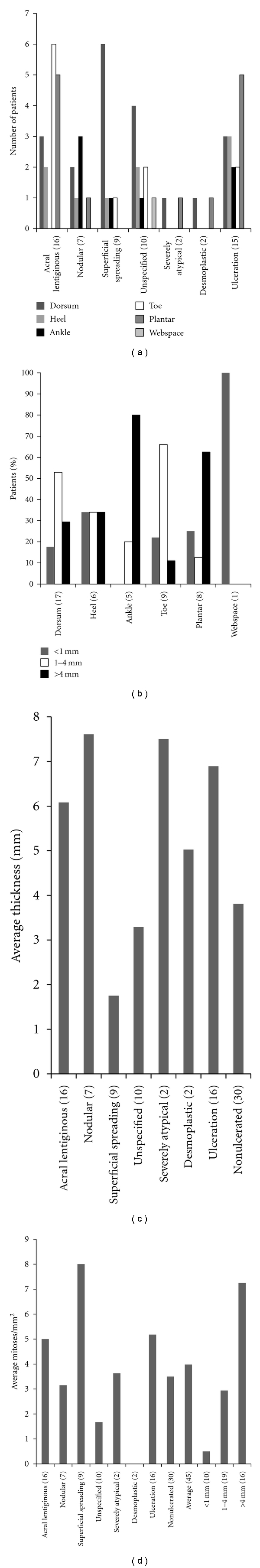
The distributions of pathology by anatomic site of origin (a), thickness by anatomic site of origin (b), thickness by pathology (c), and mitotic index by pathology and thickness (d).

The correlations between site of origin, pathologic subtype, thickness, and mitotic index were examined. Acral lentiginous, nodular, superficial spreading, unspecified, severely atypical, desmoplastic, and ulcerated melanomas were well distributed across the sites of origin (Figure 2(a)). Similarly, there was a broad distribution of lesions by thickness across the sites of origin (Figure 2(b)). The distribution of the average thickness by pathologic subtype is shown (Figure 2(c)). Superficial spreading and unspecified melanomas were thinner than other subtypes, and ulcerated lesions were thicker than nonulcerated lesions. The distribution of the average mitotic index (mitoses/10 high power fields) is shown (Figure 2(d)). In general, the thicker the melanoma, the higher the mitotic index. Superficial spreading had the highest mitotic index, while there was only a minimal difference in the mitotic index between ulcerated and non-ulcerated lesions. 

**Figure 3:**
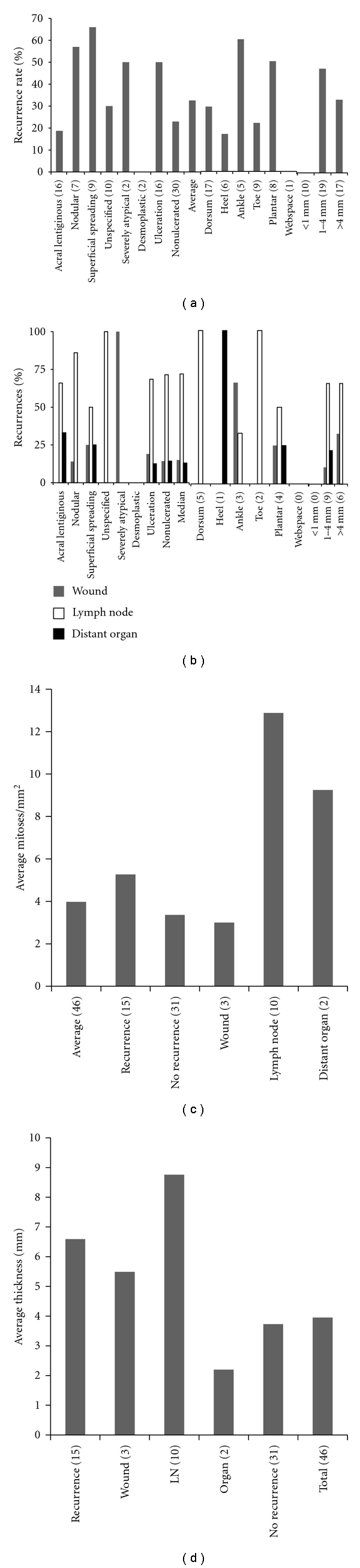
The recurrence rate by prognostic factor (a), site of recurrence by prognostic factor (b), recurrence and site of recurrence by mitotic index (c), and thickness (d).

The correlations between recurrence rate and site of origin, pathologic subtype, mitotic index, average thickness, surgical treatment, and lymph node status were examined. The rate of recurrence by histologic subtype of melanoma is shown (Figure 3(a)). There did not appear to be a correlation between recurrence rate and histology or site of origin. Similarly, there did not appear to be a correlation between location of recurrence and histology, site of origin or thickness (Figure 3(b)), or mitotic index (Figure 3(c)); thickness did not appear to correlate with location of recurrence, but lesions that recurred were thicker than those that did not recur (Figure 3(d)). The recurrence rates were 36% for wide local excision, none for toe amputation, and 100% (1/1) for below knee amputation. 65% of the patients who underwent a sentinel lymph node biopsy or lymph node dissection had positive lymph nodes. 30% of patients with positive lymph nodes developed a recurrence. There did not appear to be a correlation between recurrence rate and site of origin, pathologic subtype, mitotic index, average thickness, surgical treatment, and lymph node status.

In order to compare the recurrence rates among the different prognostic variables, the mean cumulative function (MCF) of each population was calculated. MCF assigns a cost to a population each time a recurrence occurs in that population [7–9]. The MCF plots a cost curve for a population over time [8, 9]. As more members of that population develop recurrences, more cost accrues to the population, and the curve becomes higher [8, 9]. The MCF of one population can then be compared to the MCF of another population, which thus allows for the comparison of recurrence rates between populations [7–9]. In addition to analyzing the absolute recurrence rate, MCF comparison allows for comparing how the risk of recurrence in different populations changes over time [7–9]. This method was used to evaluate how the risk of recurrence by each prognostic variable changed over time and how they were compared to each other and to the overall population as a whole.

**Figure 4:**
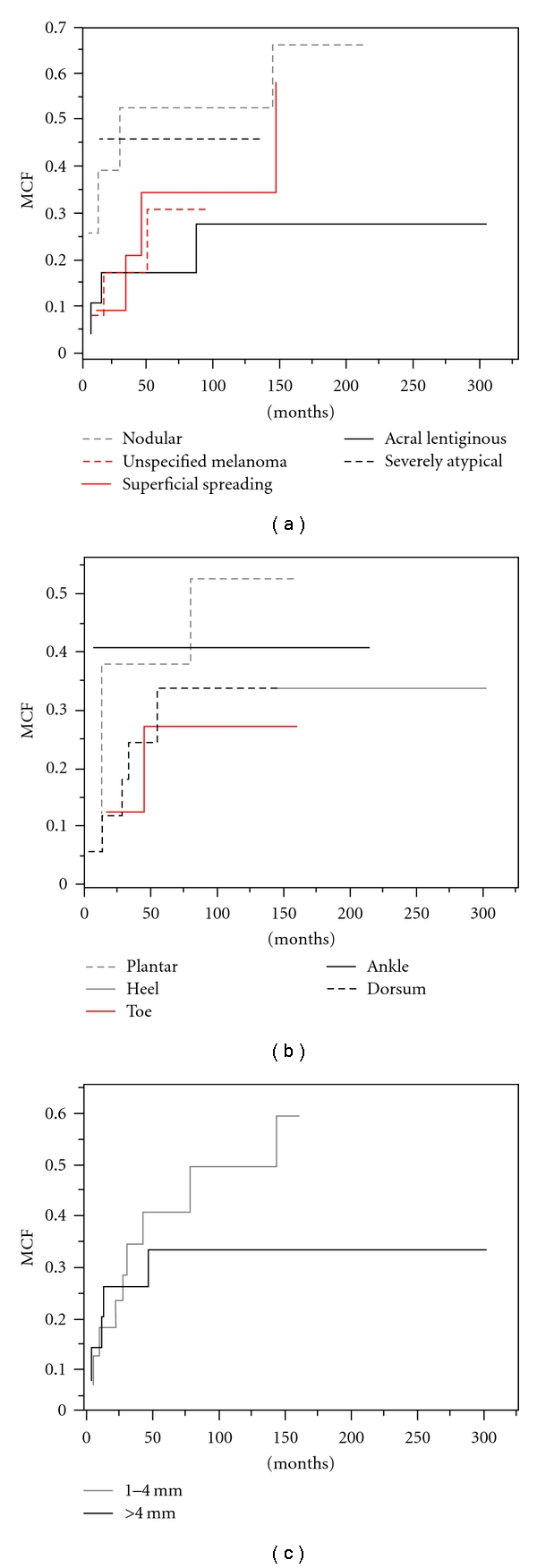
Recurrence analysis: the mean cumulative functions compared by pathology (a), anatomic site of origin (b), and thickness (c).

MCF analysis was used to compare the risk of recurrence by histologic subtype, site of origin, and thickness. The MCFs for each histologic subtype are shown (Figure 4(a)). The risks of recurrence for nodular and severely atypical melanomas were consistently high, for acral lentiginous it was consistently low, and for unspecified melanoma consistently intermediate throughout the follow-up period. For the first 150 months of followup, superficial spreading melanoma maintained a risk of recurrence similar to all subtypes combined. However, beyond 150 months, the risk of recurrence increased rapidly to approach the risk of recurrence of nodular melanoma. Neither of the patients with desmoplastic melanoma developed a recurrence and, therefore, were not included in this analysis. The MCFs for each site of origin are shown (Figure 4(b)). The risk of recurrence for plantar lesions was consistently high, for lesions on the dorsum and toe consistently intermediate, and for lesions on the ankle and heel consistently low throughout the follow-up period. The MCFs by thickness are shown (Figure 4(c)). After 25 months lesions 1–4 mm thick had a consistently high risk of recurrence, while after 75 months lesions >4 mm had a consistently low risk of recurrence. Of all the variables compared, nodular melanomas, site of origin on the plantar surface of the foot, and a thickness of 1–4 mm maintained the consistently highest risk of recurrence. Lesions <1 mm thick did not recur and therefore were not included in this analysis. Two patients died: one from pneumonia at age 103, the other from melanoma at age 72, 297 and 22 months after being diagnosed with foot melanoma, respectively. Overall survival was 96% with a 91-month median followup. 

## 4. Discussion

Foot melanoma is a rare disease which has not been adequately examined in the literature. From 1957 to 2010 only one publication examined cutaneous foot melanoma alone without confounding results by combining with subungual, hand or other lower extremity primary sites (Table 1) [2, 3, 6, 10–24]. There has not been an adequate evaluation of prognostic factors, such as histologic subtype, site of origin, or even lymph node status. Because of the great variability in reporting followup, thickness, recurrence, and survival rates, it is difficult to appropriately quantify recurrence and survival analyses or draw conclusions.

This study reviewed 46 cutaneous foot melanoma patients with a broad distribution of demographic characteristics, histology, thicknesses, mitotic indexes, and sites of origin. Although there were more cases of acral lentiginous melanoma, it was not the predominant subtype as had been historically thought of the acral sites [3]. There was a trend for more superficial spreading melanomas to arise on the dorsum of the foot (Figure 2(a)) and for thicker lesions to have a higher mitotic index, but this was without statistical significance (Figure 2(d)). There was no correlation between histology, thickness, site of origin, or mitotic index (Figures 2(a)–2(d)), which had not been previously evaluated (Table 1) [2, 3, 6, 10–24].

This study evaluated whether the absolute recurrence rate, the site of recurrence (Figures 3(a)–3(d)), or the change in recurrence rate over time correlated with prognostic factors (Figures 4(a)–4(c)), which has not been previously examined (Table 1) [2, 3, 6, 10–24]. None of the desmoplastic (2, median followup 161.4 months) or <1 mm thick lesions recurred (Figure 3(a)). There was no correlation between histology, site of origin, lymph node status, thickness, or mitotic index and overall recurrence rate (Figures 3(a)–3(d)), although thickness approached significance (*P* = 0.0946). There was no significant correlation between recurrence rate or prognostic factor and site of recurrence, but wound recurrences had the highest mitotic index and lymph node recurrences the greatest thickness (Figure 3(b)). 

 Although there was no significant correlation, there was a trend for ulcerated lesions to have a higher average thickness (Figure 2(c)) and mitotic index (Figure 2(c)) than non-ulcerated melanomas and to have twice the recurrence rate of non-ulcerated melanomas (Figure 3(a)). The implication is that ulceration in combination with thickness and mitotic index may worsen prognosis. For example, three of the four acral lentiginous melanomas that recurred were also ulcerated. Although there was no significant correlation, there was a trend for ankle and plantar melanomas to have higher recurrence rates than other sites (Figure 3(a)). Plantar melanomas accounted for one-third of all ulcerated lesions (Figure 2(a)), and over 50% of ankle and plantar lesions were >4 mm in thickness (Figure 2(b)). In contrast, only 2 of the 5 melanomas on the dorsum of the foot that recurred were ulcerated, while 3 of the 4 plantar melanomas that recurred were ulcerated. 

 Although superficial spreading melanomas had a higher absolute recurrence rate than nodular melanomas (Figure 3(a)), this did not occur until after 150 months (Figure 4(a)). The implication is that superficial spreading melanomas may have a better prognosis in terms of recurrence initially, but over time the difference disappears. This trend is an important consideration as most publications had a median followup of much less than 150 months (Table 1) [2, 3, 6, 10–24]. Acral lentiginous, unspecified melanomas, and severely atypical melanomas consistently maintained low, intermediate, and high rates of recurrences, respectively (Figure 4(a)). The implication is that other subtypes may not behave in the same way as superficial spreading melanomas, but unspecified and severely atypical melanomas were not followed as long. 

Recurrence rate by thickness changed over time, but by site of origin the rate of recurrence remained the same (Figures 4(b) and 4(c)). Although initially similar, after 75 months 1–4 mm thick melanomas had a high recurrence rate and >4 mm thick a low recurrence rate (Figure 4(c)). These data suggest that thicker melanomas recur earlier than thinner melanomas which may have implications for follow-up evaluations for both sets of patients. It should be noted that previous publications did not indicate the follow-up times for different prognostic factors or perform such recurrence analyses (Table 1) [2, 3, 6, 10–24]. The overall survival was 96% at a median followup of 91 months.

Because of the excellent survival in these patients treated over a 25-year period, no subgroup analyses could be meaningfully performed, such as molecular, genetic, or immunohistochemical analyses. In general, melanoma 5-year survival has been reported as approximately 93% for thin (<1 mm), 68% for intermediate thickness (1–4 mm), and 42% for thick lesions (>4 mm) [1]. Although it has been previously reported that melanoma in the extremity may provide a worse prognosis than other sites and that other prognostic factors, such as thickness, may predict survival better than site alone, those studies combined it with disease variants (Table 1) [2, 3, 6, 10–24]. Our results demonstrated that even foot melanoma patients with poor prognostic factors such as thickness and ulceration had an excellent survival compared to that reported for melanoma of the trunk, hands, or extremities [1–3, 6, 10–24].

In conclusion, foot melanoma remains a challenge to clinicians who must balance oncologic resection against preserving limb function. A review of the literature since 1957 reveals that there has not been an adequate analysis of prognostic factors to guide management. This study focused exclusively on patients with cutaneous foot melanoma treated over a 25-year period to comprehensively examine the prognosis and management of this rare disease. Although there were no statistically significant correlations between disease free survival and prognostic variables, only trends, survival was 96% at a median followup of 91 months. Therefore, patients with foot melanoma appear to have an excellent long-term survival even if they develop recurrent disease.

##  Conflict of Interests

The authors have no financial interests or any conflict of interests to disclose.

## Figures and Tables

**Table 1:** A review of the literature on foot melanoma that shows whether cutaneous foot melanoma exclusively, histology, site of origin on the foot, lymph node status, recurrence, overall survival, median followup, and average thickness were reported and analyzed.

Study	Foot only	Histology	Site	LNs	Recurrence	Survival	Followup (months)	Average thickness (mm)
Booher and Pack, 1957 [3]	No	No	No	Yes	Wound, regional, distant	Yes	192	No
Keyhani, 1977 [17]	No	No	No	Yes	Wound, regional, distant	Yes	60	No
Magnus, 1977 [18]	No	No	No	No	Not assessed	Yes	216	No
Feibleman et al., 1980 [12]	No	Yes	No	Yes	Wound, regional, total	No	60	No
Sondergaard and Olsen, 1980 [22]	No	Yes	Yes	Yes	Not assessed	Yes	120	No
Day Jr. et al., 1981 [11]	Yes (29)	Yes	No	No	Overall recurrence	No	60	No
Hughes et al., 1985 [16]	No	Yes	Yes	Yes	Wound, in-transit, overall	Yes	<12	No
Urist et al., 1985 [24]	No	No	No	No	Wound	No	120	No
Slingluff Jr. et al., 1990 [21]	No	No	No	Yes	Wound, regional, distant skin, other	Yes	62	2.64
Barnes et al., 1994 [10]	No	Yes	Yes	Yes	Not assessed	Yes	72	No
Fortin et al., 1995 [13]	No	No	Yes	Yes	Wound, regional, total	Yes	45	3.03
Garbe et al., 1995 [14]	No	No	No	No	Not assessed	Yes	120	2.2
Tseng et al., 1997 [2]	No	Yes	No	Yes	Wound, distant	No	67	No
Gray et al., 2006 [6]	No	Yes	No	Yes	Wound, regional, distant	No	33	1.75
Nagore et al., 2006 [19]	No	Yes	No	No	Not assessed	No	None	1.3
Soudry et al., 2008 [23]	No	No	No	Yes	Overall recurrence	Yes	53	3.28
Rex et al., 2009 [20]	No	No	No	No	Wound, satellite/in-transit, regional, distant	Yes	50	2.8
